# Development of a pro‐angiogenic stem cell‐derived vascular cell extracellular matrix‐decorated hyaluronic acid hydrogel

**DOI:** 10.1111/joa.70273

**Published:** 2026-11-04

**Authors:** Louise Hosty, Yu‐Yin Joanne Chang, Martina Konečná, Matthew McGrath, Cormac Murphy, Anja Schweikert, Mari Ozaki, Killian Hurley, Fabio Quondamatteo, Shane Browne

**Affiliations:** ^1^ Tissue Engineering Research Group, Department of Anatomy and Regenerative Medicine Royal College of Surgeons in Ireland Dublin Ireland; ^2^ CÙRAM, Centre for Research in Medical Devices University of Galway Galway Ireland; ^3^ Department of Medicine, Education and Research Centre Royal College of Surgeons in Ireland Dublin Ireland; ^4^ Trinity Centre for Biomedical Engineering Trinity College Dublin Dublin Ireland

**Keywords:** decellularised extracellular matrix, diabetic foot ulcer, hydrogel, pro‐angiogenic, wound healing

## Abstract

Diabetic foot ulcers (DFU) are debilitating chronic wounds which affect up to a third of diabetic patients. Due to the underlying diabetic pathophysiology, normal wound healing is perturbed. In particular, there is insufficient re‐vascularisation of the wound bed, ultimately leading to the development of chronic wounds. Current treatment protocols are insufficient to stimulate re‐vascularisation and wound closure, ultimately leading to ~20% of patients with a DFU requiring lower limb amputation. Extracellular matrix (ECM)‐derived biomaterials have demonstrated great promise to promote tissue vascularisation and wound repair. In the present study, the overarching goal was to develop a decellularised ECM from human induced pluripotent stem cell (hiPSC)‐derived vascular cells, and incorporate it into a hyaluronic acid‐based hydrogel for wound healing. Vascular differentiation of hiPSCs yielded a heterogenous population containing both CD31 expressing endothelial cells (iEC), and CD31‐negative stromal cells (iSC). ECM deposition by iSCs was stimulated by treatment with ascorbic acid and macromolecular crowder carrageenan. Post‐decellularisation, the ECM composition was analysed by immunofluorescent and histological staining, indicating the presence of ECM components such as fibronectin, fibrillar collagens, perlecan and glycosaminoglycans, all having implications in angiogenic processes. Decellularised ECM accelerated the capacity of iECs to form tube‐like structures when added to cell culture media in 2D. Decoration of hyaluronic acid (HyA)‐based hydrogels with this decellularised ECM enhanced metabolic activity and attachment of both HUVECs and human dermal fibroblasts seeded on these hydrogels. Taken together, this ECM‐decorated HyA‐based hydrogel has the potential to enhance angiogenesis and revascularisation in chronic wound applications such as DFU.

## Graphical abstract

This work focuses on the development of a hyaluronic acid‐based hydrogel decorated with pro‐angiogenic hiPSC‐derived vascular cell decellularised ECM, for the treatment of diabetic foot ulcers. Taken together, the results presented here show the potential of our hydrogel to enhance both angiogenesis and re‐vascularisation of wounds.

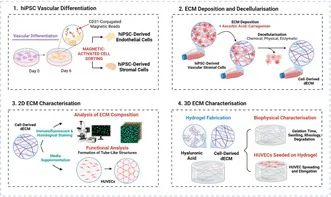

## 1 INTRODUCTION

Chronic wounds, specifically diabetic foot ulcers (DFU), are a particularly debilitating and life‐threatening complication of diabetes mellitus, affecting 19–34% of diabetic patients (Armstrong et al., 2017; McDermott et al., 2022). Due to their prevalence, they represent a significant global healthcare burden, particularly as diabetes mellitus is predicted to affect over half a billion people by 2030 (Quondamatteo, 2014). Typically, wound healing occurs in four overlapping phases: haemostasis, inflammation, proliferation and remodelling (Rodrigues et al., 2019). Timely progression through these phases ultimately leads to wound repair. However, underlying diabetic pathophysiology, including hyperglycaemia‐induced reduction of vessel contractility, blood flow and vascular endothelial growth factor (VEGF) secretion, contributes to reduced wound revascularisation, preventing tissue repair (Burgess et al., 2021; La Sala et al., 2019; Packer et al., 2023; Roberts & Porter, 2013). Delayed healing increases the exposure of wounds to the environment and can leave patients susceptible to infection and osteomyelitis (Giurato et al., 2017). Current treatments for DFUs include extensive, time‐consuming, and costly interventions such as debridement, wound dressings, pressure offloading and antibiotic therapy (Dayya et al., 2022). However, the current standard of care is clinically insufficient, ultimately leading to 20% of DFU patients eventually requiring limb amputations (McDermott et al., 2022).

Extracellular matrix (ECM)‐derived biomaterials have emerged as a promising approach to wound healing (Browne et al., 2024; Hosty et al., 2024; Ladeira et al., 2025; Liu et al., 2017; Ramos‐Rodriguez & Leach, 2025; Wang et al., 2025; Wolf et al., 2012). ECM‐derived biomaterials support accelerated wound closure, increased ECM deposition and remodelling, and revascularisation in preclinical wound models (Milan et al., 2016; Uccioli et al., 2011; Yang et al., 2020). There are a number of approaches for the development of ECM‐derived biomaterials, including the purification and processing of individual ECM components into biomaterials. Although purified ECM component biomaterials have shown promising pre‐clinical results, more complex biomaterials with an ECM profile that more closely resembles the native tissue may be necessary to support cell interactions and drive wound healing (Jeschke et al., 2005; Rho et al., 2006; Shakouri‐Motlagh et al., 2017; Taddeucci et al., 2004). Similarly, animal or human cadaver tissues have been decellularised and processed to produce both acellular grafts and ECM‐decorated biomaterials. However, strictly optimised decellularisation protocols are an essential consideration that must be made in order to mitigate immunogenic responses (Hsieh et al., 2020; Milan et al., 2016). An alternative approach involves decellularisation of in vitro cell‐deposited ECM (dECM), and its incorporation within biomaterials (Chen et al., 2022; Doherty et al., 2024; Santarella et al., 2020). The production of in vitro‐deposited ECM‐derived biomaterials is a highly tuneable and specific technique. For example, specific cell types and microenvironmental conditions can be used to drive the deposition of distinct ECM profiles and to subsequently drive specific cell behaviours (Satyam et al., 2020). Human‐induced pluripotent stem cell (hiPSC)‐derived cells offer a promising source of cell‐derived ECM (De Pieri et al., 2022; Doherty et al., 2024; Guo et al., 2023), and were therefore employed as a source of ECM in the present study. They have previously shown to have an increased capacity to deposit ECM that is enriched in components that drive vascularisation and wound healing, for example, collagens and fibronectin (Clayton et al., 2018; McDermott et al., 2022; Santarella et al., 2020; Shamis et al., 2012). Similarly, their capacity for unlimited expansion can be leveraged to allow the scaling‐up of ECM deposition as a novel therapeutic approach (Cerneckis et al., 2024).

Hydrogels are hydrophilic polymer networks which can be crosslinked in situ to fill irregularly shaped defects (Browne & Healy, 2019; Hosseini et al., 2025; Monaghan et al., 2023). The biophysical properties of hydrogels, including their hydrophilic nature, allow for the absorption and retention of wound exudate, facilitating the retention of moisture and growth factors which are essential for healing at the wound site (Li et al., 2020; Shan & Wu, 2024; Zhang, Yao, et al., 2021). Hyaluronic acid (HyA)‐based hydrogels in particular have received much attention for wound healing applications (Zhang, Li, et al., 2021). HyA is a non‐sulphated glycosaminoglycan (GAG) which is present in the native ECM and is inherently biocompatible (Della Sala et al., 2022; Petit, Chang, et al., 2025). HyA has been shown to stimulate vascularisation through endothelial cell surface receptor‐mediated signal transduction (CD44 and RHAMM) (Slevin et al., 2002). In the current study, HyA was chemically modified by the addition of acrylate groups (AcHyA), allowing for stable hydrogel formation by addition of dithiol crosslinkers as previously described (Browne et al., 2020).

The present work aims to develop a pro‐angiogenic hydrogel decorated with dECM generated by hiPSC‐derived cells. To establish a scalable and pro‐angiogenic source of ECM, hiPSCs were differentiated towards a vascular lineage, giving rise to hiPSC‐derived vascular stromal cells (iSC). iSCs were cultured for 21 days in the presence of a macromolecular crowder which has been demonstrated to enhance and accelerate ECM deposition. Following decellularisation, the capacity of dECM to drive angiogenesis was demonstrated. First, we identified a range of components present within the dECM which are known to be pro‐angiogenic and support wound healing. Similarly, we showed the capacity of this dECM to drive the formation of vascular‐like structures in 2D, and to support the angiogenic behaviour of cells when seeded on dECM‐decorated AcHyA hydrogels.

## 2 MATERIALS AND METHODS

### 2.1 Vascular differentiation of hiPSCs to generate vascular stromal cells

#### 2.1.1 hiPSC line generation

The iPSC line RCSI‐D1 was generated according to Ozaki et al., under review. Briefly, blood samples were taken from a healthy male donor and peripheral blood mononuclear cells (PBMCs) were isolated using SepMateTM PBMC Isolation tubes (STEMCELLTM Technologies) per manufacturer's instructions. Reprogramming was carried out using the CytoTune™‐iPS 2.0 Sendai Reprogramming Kit (Invitrogen™) with minor adaptations. hiPSCs were characterised for pluripotency via immunofluorescence (IF) staining for SSEA‐4, TRA‐1‐60 and TRA‐1‐81, and a normal karyotype was confirmed via Karyostat Assay (Thermo Fisher Scientific).

#### 2.1.2 hiPSC culture

hiPSCs (RCSI‐D1) were cultured in mTESR™ Plus media (100‐0276; StemCell Technologies, EU), on Corning™ Matrigel™ hESC‐Qualified Matrix (11,573,560; Fisher Scientific, IE)‐coated plates. mTESR™ Plus was replaced every 1–2 days.

#### 2.1.3 hiPSC vascular differentiation

Vascular differentiation of hiPSCs was performed as previously described (Browne et al., 2020). Briefly, hiPSCs were seeded 2–3 days prior to differentiation. When cells reached ~70% confluence, differentiations were initiated by replacing the media with LaSR media (Advanced Dulbecco's Modified Eagle Medium (12,491,023, Sigma‐Aldrich, DE), 60 μg/mL ascorbic acid (AA) and 2.5 mM GlutaMAX 100× [35,050,061; Fisher Scientific, IE]) with 10 μM CHIR99021 (4423/10; R&D Systems; distributed by Bio‐Techne, IE) for mesoderm induction. On day 2 and day 4, media was replaced with LaSR media supplemented with 50 ng/mL VEGF (BT‐VEGF‐020; R&D Systems; distributed by Bio‐Techne, IE) for vascular specification.

### 2.2 Magnetic‐activated cell sorting (MACS)

On day 6 of the differentiation protocol, cells were sorted based off CD31 expression into two distinct populations (CD31+ cells and CD31‐ cells). Cells were incubated with trypsin at room temperature for 4 min, then quenched and centrifuged in 2% Fetal Bovine Serum (FBS) at 300 g for 5 min. Cells were subsequently resuspended in CD31 (1:50) (PECAM‐1) monoclonal antibody (WM‐59) (WM59) (14–0319‐82; Fisher Scientific, IE) with 2% FBS and left on a shaker at room temperature for 1 h. The cell suspension was then washed in phosphate buffered saline (PBS) with 2% FBS and resuspended in DynaBeads™ goat‐anti mouse (Fisher Scientific, IE) in 2% FBS. Cells were left rocking for 1 h at room temperature and sorted using a magnetic stand (DynaMag™‐15 Magnet, Fisher Scientific, IE). CD31‐ cells (iSCs) were cultured in endothelial growth media (EGM‐2) (C22111; PromoCell GmbH, Germany; distributed by Brennan & Co., IE) + 10 μM Y27632 dihydrochloride (1254; TOCRIS; distributed by Bio‐Techne, IE) and CD31+ (iECs) cells were cultured in EGM‐2 + 10 μM Y27632 dihydrochloride + 10 μM SB431542 (1614; TOCRIS; distributed by Bio‐Techne, IE). Media containing Y27632 dihydrochloride was removed after 24 h and replaced with fresh EGM‐2.

### 2.3 Biological characterisation of differentiated cells

#### 2.3.1 Immunofluorescence staining of iSCs and iECs

Cells were fixed for 30 min in 10% neutral buffered formalin and blocked for 1 h in 1% bovine serum albumin (BSA), and permeabilised for 15 min in 0.1% Triton X‐100 where necessary. Primary antibodies CD31 (PECAM‐1) (1:500), MYH11 (1:100; HPA015310; Merck Life Sciences, DE), SM‐22α (1:100; MA547914; Fisher Scientific, IE) and vimentin (1:100; Fisher Scientific, IE) were diluted in 1% BSA and incubated overnight at 4°C. Cells were then washed three times in PBS, and secondary antibodies goat anti‐mouse‐488 (1:2000; 10,256,302; Fisher Scientific, IE) and donkey anti‐rabbit‐647 (1:1000; A31573; Fisher Scientific, IE) were diluted in PBS and incubated at room temperature for 2 h. Cells were then stained with DAPI for 15 min, before washing with PBS and imaging using a Zeiss Examiner.Z1 confocal microscope. Appropriate controls were included for the validation of staining, including unstained and secondary antibody‐only stained samples.

Quantification of the co‐localisation of immunofluorescent staining with cell nuclei was carried out in FIJI to quantify the composition of cell types in the iSC population, and to confirm their stromal‐like nature. Image channels were separated and the background was subtracted to remove non‐specific signals. Otsu threshold was used to define regions of interest, and binary masks were generated to quantify nuclear overlap with immunofluorescent signals of interest.

#### 2.3.2 Functional analysis of iECs by Matrigel tube formation assay

A Matrigel™ tube formation assay was carried out as previously described (Palomeque Chávez et al., 2025). Briefly, 96‐well plates were coated with 80 μL of Corning™ Matrigel™ Growth Factor Reduced Basement Membrane Matrix (356,230; Fisher Scientific, IE) and incubated at 37°C for 30 min. iECs were seeded in EGM‐2 at 10,000 cells/well of a 96‐well plate and imaged hourly using a Zeiss Cell Discoverer 7 microscope over 48 h. The Angiogenesis Analyser plugin for ImageJ was used to quantify the number of tubes, junctions, branches and branching length (Carpentier et al., 2020).

### 2.4 iSC ECM deposition and collection

#### 2.4.1 Culture of iSC with macromolecular crowders for the deposition of ECM

iSCs were seeded at 13,000 cells/cm^2^ in EGM‐2 in T75 flasks. On day 1, cells were supplemented with 100 μg/mL ascorbic acid (AA) or 100 μg/mL AA and 50 μg/mL carrageenan (C) (Zeugolis, 2021). Media were refreshed every 3–4 days for 21 days. On day 21, media were aspirated, and cells were decellularised.

#### 2.4.2 AlamarBlue metabolic activity assay

Media was replaced with 10% AlamarBlue reagent in EGM‐2 (10,161,053; ThermoFisher, IE), and cells were incubated for 2 h at 37°C. Subsequently, 100 μL of media + AlamarBlue solution from each well was added in triplicate to a black 96‐well plate, and fluorescence was read using a TECAN Plate Reader at 545/590 nm.

#### 2.4.3 Decellularisation of the cell monolayer

At day 21, the cell monolayer was washed with deionised water three times and underwent three freeze–thaw (F/T) cycles at −20°C, prior to incubation in a decellularisation solution (20 mM ammonium hydroxide and 0.5% Triton X‐100 in water) for 1 h, rocking at room temperature. Samples were washed three times with deionised water and centrifuged at 150 g for 5 min each time. Samples were then treated with 10 μg/mL DNAse I for 1 h at 37°C, and washed a further three times with deionised water by centrifugation at 150 g for 5 min each time. Samples were then stored at −20°C until lyophilisation at −58°C and 0.1 mBar.

#### 2.4.4 DNA quantification

Samples were lysed with lysis buffer (0.2 M sodium carbonate (Na2CO3) + 1% Triton X‐100 in dH2O), and stored at −20°C, prior to three F/T cycles. Subsequently, DNA was measured using the manufacturers' Standard Operating Procedure Quant‐iT Kit for DNA quantification (10,545,213; Fisher Scientific, IE). Samples and DNA standards were plated in duplicate to a black 96‐well plate, and fluorescence was read at 480/520 nm using a TECAN Plate Reader. Samples were compared with a standard curve of 0–2000 μg/mL DNA stock.

#### 2.4.5 dECM solubilisation

After lyophilisation, dECM was digested in 3 mg/mL pepsin from porcine gastric mucosa (lyophilised powder, ≥3200 units/mg protein P6887; Merck Life Sciences, DE) in 1 M acetic acid and left shaking on a ThermoMixer (Eppendorf) at 37°C until solubilised. The pH was then neutralised using 1 M NaOH and pH indicator paper.

After solubilisation, dECM samples were diluted in PBS 1:20. Micro BCA™ Protein Assay Kit (23,235; ThermoFisher, IE) was used to quantify protein concentration per sample. The assay was carried out according to the manufacturer's instructions, and protein concentration was determined by comparison with a BSA standard curve. Absorbances were read at 562 nm using a plate reader.

### 2.5 Immunofluorescent and histological staining of ECM and dECM, and analysis of its protein content and pro‐angiogenic capacity

#### 2.5.1 Histological assessment of iSC‐deposited ECM and dECM

Alcian blue staining was carried out for the detection of both GAGs and sulphated (s)GAGs within the ECM. Samples were fixed for 30 min in 10% neutral buffered formalin and washed three times with PBS. Samples were then washed once with deionised water, followed by staining with alcian blue solution at pH 2.5 (100 mL 3% glacial acetic acid in deionised water, 1 g alcian blue) and pH 1 (10 mL hydrochloric acid in 90 mL deionised water, 1 g alcian blue) for 30 min. Samples were then washed three times with tap water and counterstained with haematoxylin for 10 min prior to more washes with tap water until it ran clear. Samples were then dehydrated using 70%, 95% and 100% ethanol and fixed with distrene plasticiser xylene (DPX) for imaging.

Fibrillar collagens were stained with picosirius red solution (1 g/L Sirius red F3B (CI 35,782) in saturated aqueous solution of picric acid) for 1 h. Samples were then washed twice with 0.5% acetic acid, followed by PBS. Finally, samples were stained with 4′,6‐Diamidino‐2‐Phenylindole (DAPI) for 15 min, and imaged using a fluorescent microscope.

#### 2.5.2 Immunofluorescent staining of pro‐angiogenic components in the dECM

Cells were fixed for 30 min in 10% neutral buffered formalin and blocked for 1 h in 1% BSA, and permeabilised for 15 min in 0.1% Triton X‐100 where necessary. Primary antibodies fibronectin (1:500; 14‐9869‐82; ThermoFisher, IE) and perlecan (1:100; 17,760,594; Fisher Scientific, IE) were diluted in 1% BSA, and incubated overnight at 4°C. Cells were then washed three times in PBS, and secondary antibodies goat anti‐mouse‐488 (1:2000; 10,256,302; Fisher Scientific, IE) and donkey anti‐rabbit‐647 (1:1000; A31573; Fisher Scientific, IE) were diluted in PBS, and incubated at room temperature for 2 h. Cells were then washed with PBS three times, and imaged using a Zeiss Examiner.Z1 confocal microscope. Appropriate controls were included for the validation of staining, including unstained and secondary antibody‐only stained samples.

#### 2.5.3 Analysis of the capacity of dECM to drive the formation of tube‐like structures in 2D

A Matrigel™ tube formation assay was carried out as described above. Briefly, 10,000 cells were seeded per well of a 96‐well plate in EBM (negative control), or in 0.05 mg/mL dECM in EBM. Cells were imaged hourly using a Zeiss Cell Discoverer 7 microscope over 48 h. The Angiogenesis Analyser plugin for ImageJ was used to quantify the number of tubes, junctions, branches and branching length (Carpentier et al., 2020). Quantification of vascular structures (junctions and tubes) was carried out using the Angiogenesis Analyser for ImageJ (Carpentier et al., 2020).

### 2.6 Development and biophysical analysis of HyA hydrogels decorated with dECM

#### 2.6.1 Modification of HyA with acrylate groups

AcHyA was synthesised using a previously published protocol (Browne et al., 2020; Jha et al., 2016). Briefly, 3 mg/mL HyA sodium salt (301 K‐499 KDa; Lifecore Biomedical, USA) was reacted with adipic dihydrazide (ADH; A0638; Sigma‐Aldrich, DE) to add hydrazide groups to HyA (HyA‐ADH), followed by 3 days of dialysis in SnakeSkin™ dialysis membrane (10,000 MWCO; Invitrogen) and precipitation in ethanol. N‐Acryloxysuccinimide (NAS; A8060; Sigma‐Aldrich, DE) was reacted with HyA‐ADH to graft acrylate groups on HyA, followed by 3 days of dialysis and lyophilisation at −58°C and 0.1 mBar for 3 days. Functionalisation of HyA with acrylate groups was confirmed by proton Nuclear Magnetic Resonance (^1^H‐NMR) spectroscopy.

#### 2.6.2 Formation of AcHyA hydrogels

AcHyA hydrogels were formed by dissolving 3 wt% AcHyA in triethanolamine‐buffer (TEOA; 0.2 M, pH 8; Invitrogen) for 90 min. This was followed by chemical crosslinking via reaction between thiol‐acrylate groups using 1.5 wt% PEG‐dithol (704,539; Sigma‐Aldrich, DE) and 0.05 or 0.5 mg/mL dECM in TEOA buffer. Hydrogels were weighed prior to swelling and degradation assays.

#### 2.6.3 Degradation

HyA‐based hydrogels were weighed and incubated in PBS for 24 h, and re‐weighed prior to the addition of 10 U/mL of hyaluronidase (H3506; Sigma‐Aldrich, DE). Hydrogels were blotted to remove excess liquid, weighed and the hyaluronidase solution refreshed at regular intervals.

### 2.7 Cumulative protein release from dECM‐decorated hydrogels

AcHyA‐based hydrogels were incubated in PBS for 24 h, and subsequently suspended in 10 U/mL of hyaluronidase (H3506; Sigma‐Aldrich, DE). After 24 h and 72 h the degradation supernatant was collected and stored at −20°C. Protein release from the dECM‐decorated hydrogels was assessed by micro‐BCA™ assay. Non‐loaded hydrogels were used as assay blanks to account for hyaluronidase release/background signals. Micro‐BCA™ assay was carried out as described above.

#### 2.7.1 Mechanical testing

The viscoelastic properties of AcHyA‐based hydrogels as a function of dECM incorporation were measured using an oscillatory HR‐1 Discovery Hybrid Rheometer with a parallel plate 8 mm geometry. Gelation time was measured by mixing the PEG‐dithiol crosslinker with the AcHyA macromer solution and immediately placing the mixture on the rheometer and determined as the time at which the storage modulus crosses the loss modulus at 0.1% strain and 1 Hz. Storage modulus was measured 90 min post‐crosslinking at 0.1% strain at 1 Hz for 5 min. Young's modulus was studied using a Zwick mechanical tester (Zwick/Roell, Ulm, Germany). A 5 N load cell was used, and Young's modulus was calculated by the slope of the linear portion of the stress–strain curve from 0 to 10% strain.

### 2.8 Analysis of the biological capacity of AcHyA decorated with dECM

#### 2.8.1 Cell culture

Human Umbilical Vein Endothelial Cell (HUVEC) were cultured in EGM‐2 + 1% penicillin–streptomycin. Media was refreshed every 2–3 days, and passaged when HUVECs reached ~80% confluence. Experiments were carried out with HUVEC between passages 5 and 7.

Human Derived Fibroblasts (hDF) were cultured in low glucose Dulbecco's Modified Eagles Medium (DMEM) with 10% FBS and 1% penicillin–streptomycin. Experiments were carried out with HDFs between passages 5 and 10.

#### 2.8.2 Seeding of cells on AcHyA hydrogels decorated with dECM

HyA‐based hydrogels, with or without the addition of dECM, were formed in cell culture inserts (PICM01250; Merck, DE). 100,000 cells (HUVEC or hDF) were then seeded on each HyA‐based hydrogel. Metabolic activity of cells seeded on HyA‐based hydrogels was measured on days 3 and 7 using the AlamarBlue assay.

#### 2.8.3 Fluorescent staining of HUVECs and HDFs to assess cell morphology and spreading

On day 7 of culture, media was removed, and cell‐seeded AcHyA hydrogels were washed with PBS. Samples were fixed in 10% formalin for 2 h, and stained with phalloidin (1:400; A12380; Invitrogen™). Samples were then stained with 1:5000 DAPI for 20 min, and imaged using a Zeiss Examiner.Z1 confocal microscope.

#### 2.8.4 Assessment of the pro‐angiogenic capacity of dECM‐decorated hydrogels in 3D

To determine the pro‐angiogenic effect of dECM‐decorated hydrogels on cells, a Matrigel™ tube formation assay using conditioned media from HDFs seeded on AcHyA hydrogels was carried out. The supernatant from hDFs seeded on dECM‐decorated hydrogels was collected at day 7. Supernatant was stored at −20°C until the time of assay. Controls included DMEM with 10% FBS as a negative control, and EGM‐2 as a positive control. Cells were imaged hourly using a Zeiss Cell Discoverer 7 microscope over 48 h. Tube formation was calculated using the Angiogenesis Analyser for ImageJ (Carpentier et al., 2020).

### 2.9 Statistical analysis

All quantitative measurements were performed at *n* = 3. All values represent mean ± standard deviation (SD), unless otherwise stated. One‐way ANOVA with Tukey's or Dunnett's post hoc test was performed when one treatment was compared. Two‐way ANOVA with Dunnett's or Sìdàks post hoc test was performed when one treatment was compared across two factors. Normality of the pooled residuals was assessed using both visual inspection of Q‐Q plots and Shapiro–Wilk analysis, given the small sample sizes. *p* < 0.05 was used to define statistical significance. Statistical analysis was performed using GraphPad Prism 10.6.

## 3 RESULTS

### 3.1 Vascular differentiation of hiPSCs

After vascular differentiation of hiPSCs, cells were separated by CD31 expression using MACS, yielding two cell populations: CD31+ iECs and CD31‐ iSCs (Figure 1a). On day 0 of the differentiation protocol, hiPSCs are present in distinct colonies (Figure 1b) and gradually become a heterogenous population of cells after mesoderm induction (Figure 1c) and vascular specification (Figure 1d). Prior to MACS on day 6, there is widespread, non‐uniform expression of CD31, indicating successful vascular differentiation of hiPSCs and the presence of a heterogenous cell population (Figure 1e).

**FIGURE 1:**
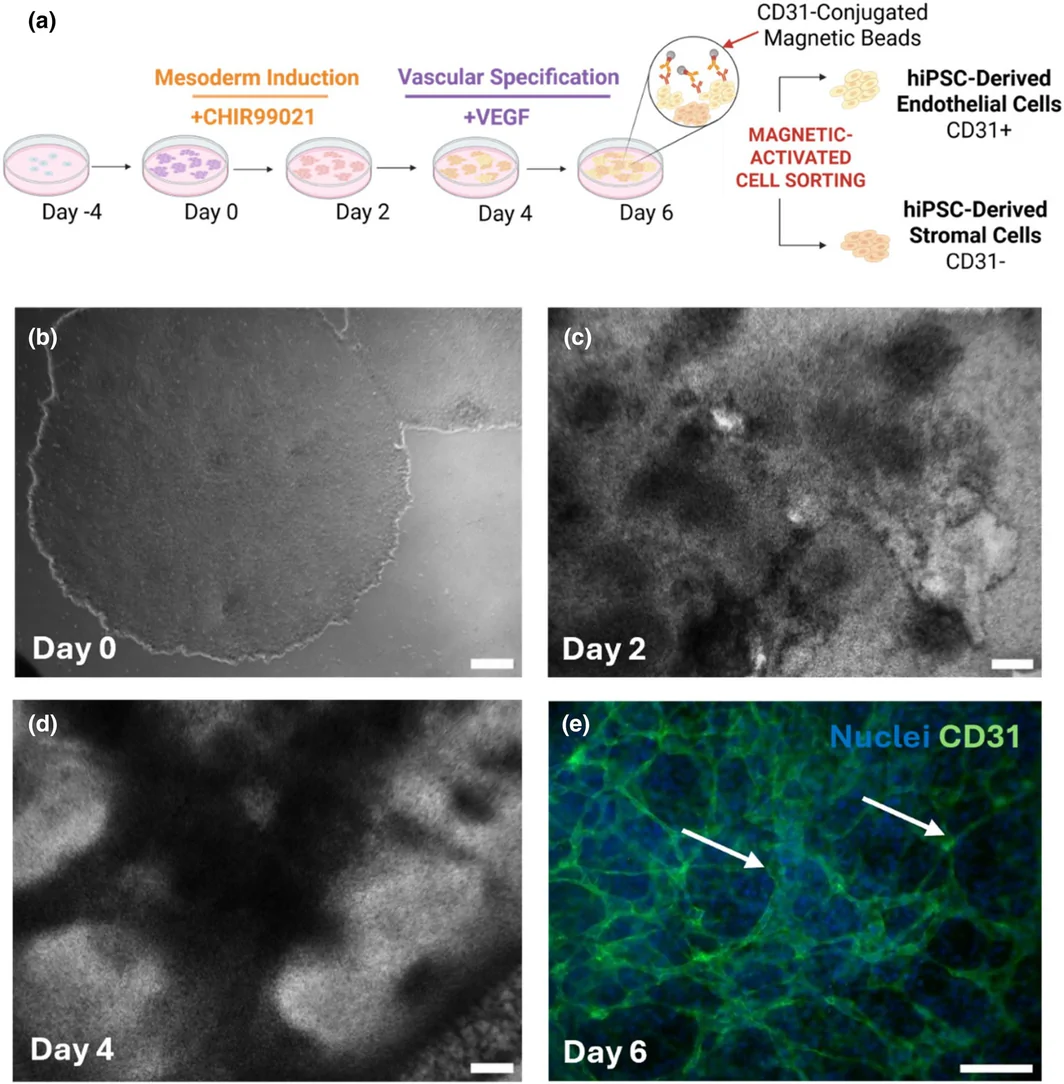
hiPSCs are differentiated towards a vascular lineage. (a) hiPSC differentiation protocol schematic. Figure created in BioRender. (b–d) Brightfield images showing hiPSCs become a heterogenous population of cells, which exist in a web‐like morphology at days 4–6. Scale bar 300 μm. (b) hiPSCs are cultured for 3–4 days until confluent. On day 0, cells are supplemented with CHIR99021 for 2 days. (c, d) On day 2 and day 4, cells are supplemented with VEGF. (e) CD31 (green)/DAPI (blue) immunofluorescent staining on day 6, showing successful vascular differentiation of hiPSCs. CD31 staining is also indicated by arrows. Scale bar 200 μm.

### 3.2 Characterisation of hiPSC‐derived iSCs and iECs

Each cell population was assessed to determine the presence of various phenotypic markers. iSCs (Figure 2a) demonstrated no CD31 expression (Figure 2b), indicating the successful separation of iSCs from iECs, and that there were no endothelial cells present in the population post‐sorting. iSCs stained positively for MYH11 (Figure 2c), SM‐22α (Figure 2d) and vimentin (Figure 2e), indicating their stromal‐like nature. Expression of SM‐22α and vimentin is typically seen in cells such as smooth muscle cells, fibroblasts and pericytes. Quantification of these stains (Figure 2f) indicated 59.8 ± 12.2% MYH11 expression, 25.7 ± 26.4% SM‐22α expression, 55.5 ± 27.4% vimentin expression and 0% CD31 expression, indicating that fibroblasts and smooth muscle cells occupy approximately half of the cell population, respectively. iECs were characterised by positive CD31 immunofluorescent staining (Figure 2g) and the formation of tube‐like structures over 1 to 3 h (Figure 2h,i).

**FIGURE 2:**
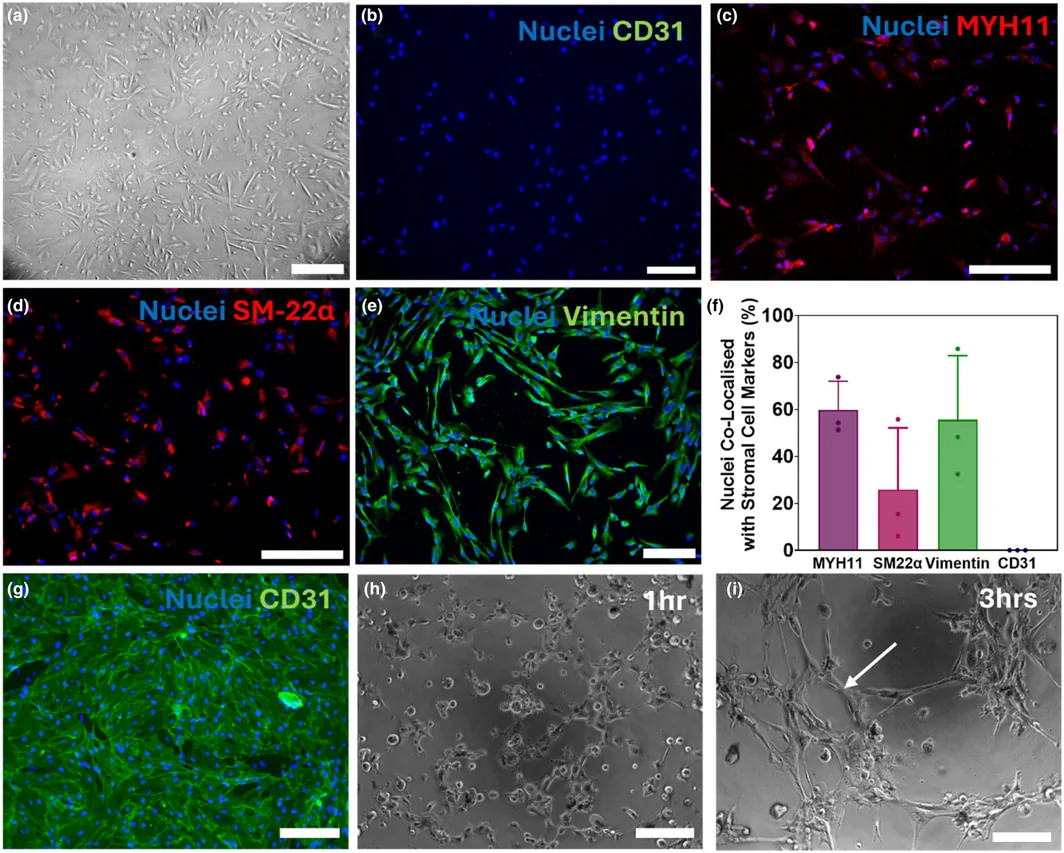
hiPSC vascular differentiation yields a heterogenous population of CD31 expressing hiPSC‐derived endothelial cells (iECs), and CD31‐negative hiPSC‐derived stromal cells (iSCs). (a) Brightfield image of iSCs. Immunofluorescent staining of iSCs indicating (b) negative CD31, (c) MYH11 (red), (d) SM‐22α (red) and (e) vimentin (green, also indicated by arrow) expression. (f) Quantification of the colocalisation of key vascular stromal cell markers with nuclei, quantifying the expression of MYH11, SM22α, vimentin and CD31 in the iSC population. Data represents mean ± SD, *n* = 3 technical replicates with imaging replicates in triplicate. (g) Immunofluorescent staining of iECs indicating CD31 expression (green), and (h, i) the formation of tube‐like structures by iECs from 1 to 3 h. Tube‐like structure indicated by arrow. Scale bar 200 μm.

### 3.3 A combination of physical, chemical and enzymatic treatments successfully decellularises cell monolayer and retains ECM deposited by iSCs

In the present study, the macromolecular crowder carrageenan (C) and ascorbic acid (AA) were added to the culture media to accelerate ECM deposition. Following 21 days of culture with AA and C (Figure 3a), no effect was observed on iSC metabolic activity (Figure S1A). The yield of ECM as a function of the addition of AA and C was assessed after decellularisation (dECM) and lyophilisation of the collected ECM. Measurement of the dECM indicated that treatment of iSCs with AA+C at 13,000 cells/cm^2^ and 2% serum resulted in the highest dECM yield (Figure S3A–C).

**FIGURE 3:**
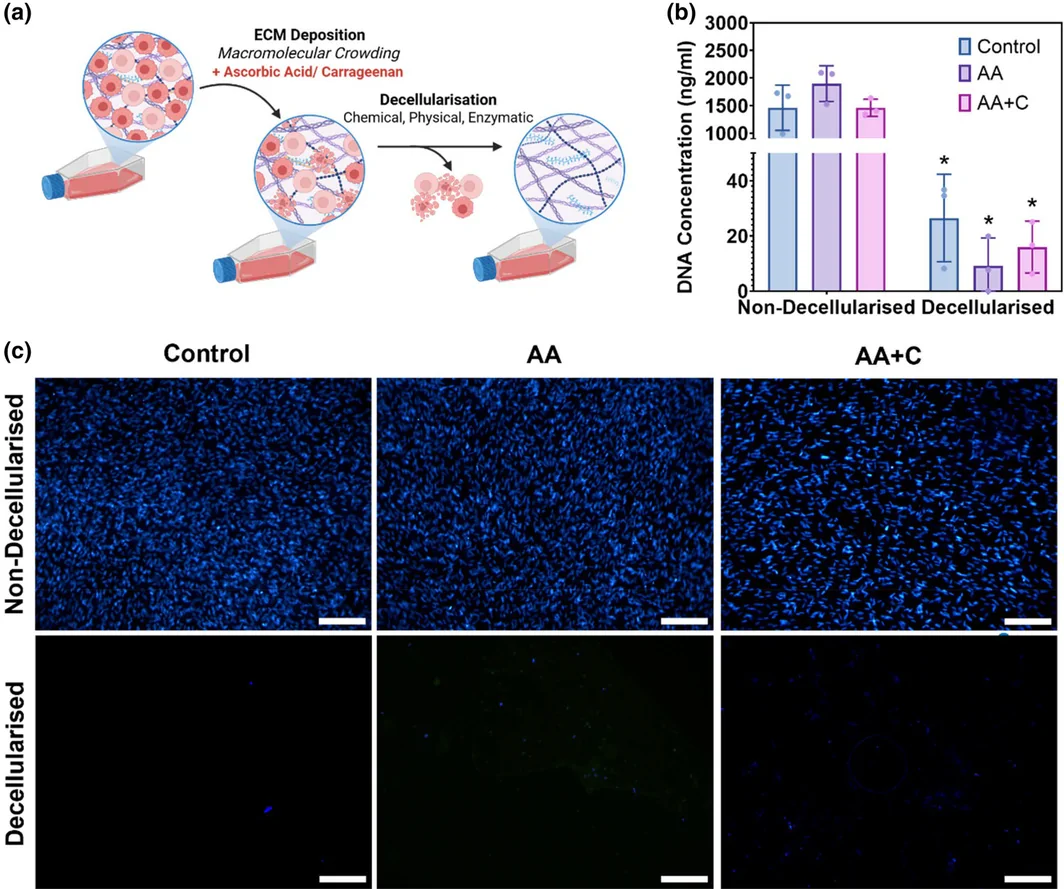
Cells are successfully removed after treatment with a combination of mechanical, physical and enzymatic decellularisation methods. (a) Schematic of ECM deposition by iSCs and subsequent decellularisation. Cells are cultured in 2D for 21 days, with supplementation of AA or AA+C. Samples are then decellularised using water, 3× F/T cycles, ammonium hydroxide, triton X‐100 and DNAse, to obtain cell‐derived ECM. Figure created in BioRender. (b) Quantification of DNA concentration in non‐decellularised and decellularised samples shows a significant reduction in DNA concentration, indicating successful decellularisation. Data represents mean ± SD. Analysis performed by two‐way ANOVA with Sìdàks multiple comparisons test; *n* = 3 technical replicates with assay replicates in triplicate, *p < 0.05 in comparison with corresponding non‐decellularised sample. (c) Strong reduction in DAPI staining of cell nuclei after decellularisation further backs up the successful removal of cells. Scale bar 100 μm.

For decellularisation, cells were initially treated with 20 mM ammonium hydroxide, 0.5% triton X‐100 and deionised water; however, there was minimal reduction observed in DNA concentration and DAPI staining (Figure S2A,B). Subsequently, the addition of 3 F/T cycles and DNAse treatment to the protocol led to a significant reduction in DNA concentration, with quantification indicating a reduction from 1461.8 ± 412.5 to 26.5 ± 15.9 ng/mL in control cells, 1897.7 ± 323.4 to 9.2 ± 10 ng/mL in AA‐treated cells and 1460 ± 154.3 to 15.97 ± 9.4 ng/mL in AA+C‐treated cells (Figure 3b). These reductions represent approximately a 98.9% reduction in DNA concentration across all samples. DAPI staining further verified the efficient removal of cells by the decellularisation approach used (Figure 3c).

### 3.4 Immunofluorescent and histological staining of the ECM indicated the presence of a range of pro‐angiogenic components

As a result of iSCs which were cultured in media containing AA+C yielding the greatest amount of dECM, this group was selected for further analysis. Immunofluorescent and histological staining were carried out to analyse the composition of ECM pre‐ and post‐decellularisation. Fibronectin‐, fibrillar collagen‐ and perlecan‐rich matrices were observed in AA+C‐ECM (Figure 4a). Similarly, AA+C‐ECM displayed strong alcian blue staining at both pH 1 and 2.5, indicating the presence of both GAGs and sulphated GAGs (sGAGs) respectively (Figure 4b). Similar ECM profiles were observed in control‐ECM and AA‐ECM (Figure S4A,B). Following decellularisation, fibronectin, fibrillar collagens and perlecan were retained to a larger extent than both GAGs and sGAGs (Figure 4a,b). Similarly, fibronectin, fibrillar collagens and perlecan appeared to be retained to a larger extent in AA+C‐dECM than control‐ and AA‐dECM (Figure S4A). Prior to the decoration of hydrogels with dECM, dECM was lyophilised (Figure 4c) and further solubilised to ensure both its bioactivity and homogeneity within the hydrogels. Post‐solubilisation, a BCA assay determined the protein concentration per dECM sample, indicating that they contain ~65% protein (Figure 4d).

**FIGURE 4:**
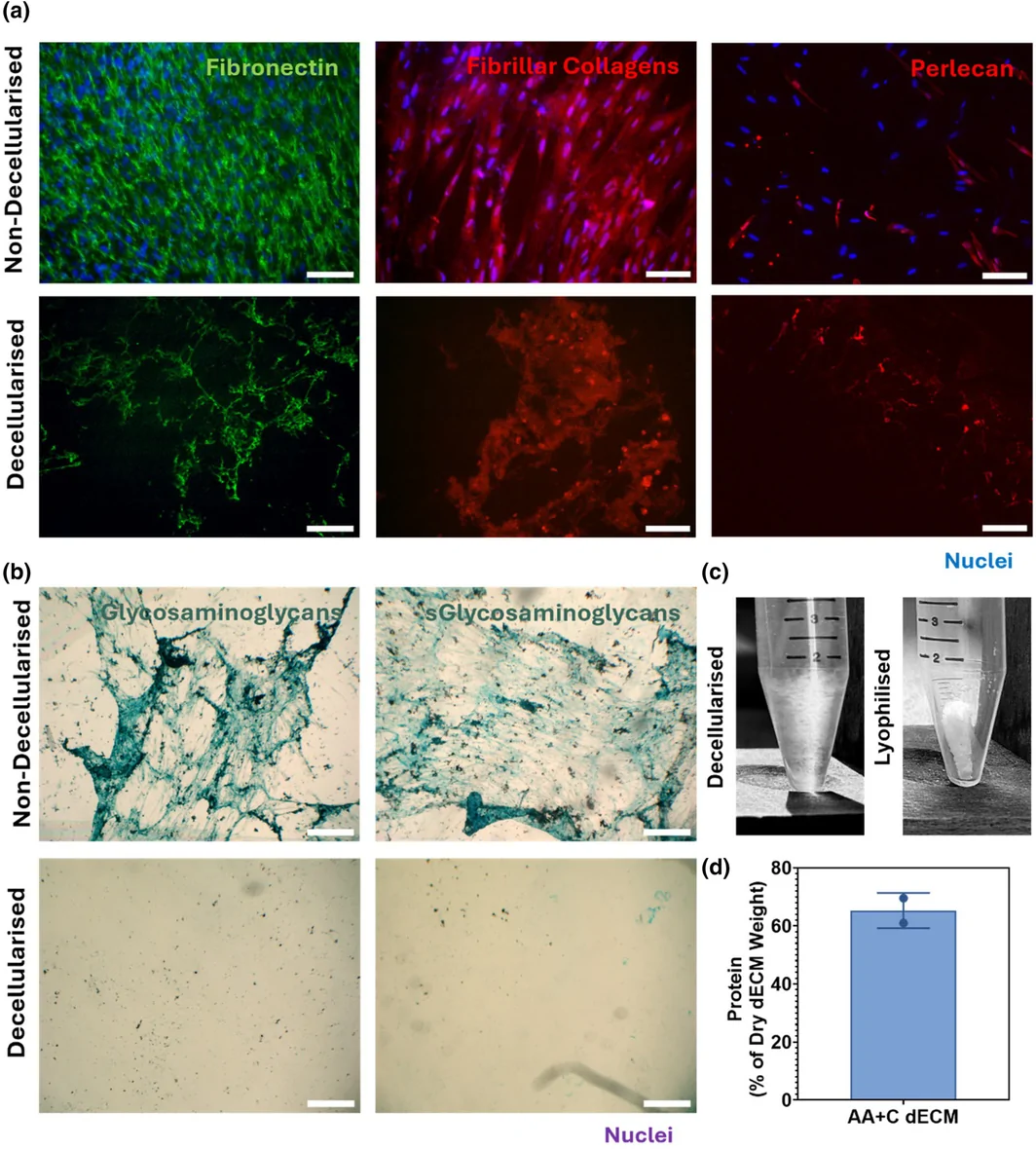
iSC‐deposited ECM contains fibronectin, fibrillar collagens, perlecan, GAGs and sGAG. (a) Immunofluorescent and histological staining of iSC‐deposited ECM and dECM supplemented with AA and C over 21 days, shows the deposition and retention of fibronectin (green), fibrillar collagens (red) and perlecan (red). Scale bar 200 μm for fibronectin, fibrillar collagens and perlecan. (b) Histological staining of alcian blue at pH 1 and 2.5 indicate the deposition and retention of GAGs and sGAGs. Alcian blue staining also indicated by arrows at both pH 1 and 2.5. Scale bar 500 μm for GAGs and sGAGs. (c) Representative images of decellularised ECM prior to and after lyophilisation. (d) Protein content in dECM samples, normalised to dECM mass post‐lyophilisation. *n* = 2 technical replicates, with assay replicates performed in triplicate.

### 3.5 dECM enhances iEC tube formation in 2D

A Matrigel™ tube formation assay was used to assess the effect of dECM media supplementation on the formation of tube‐like structures by iECs (Kubota et al., 1988). An increase in the organisation of iECs into tube‐like structures in response to dECM treatment is shown in Figure 5a. The formation of tube‐like structures (Figure 5b) and junctions (Figure 5c) was transiently accelerated over the first 2 h. The early increase in the number of junctions formed by iECs treated with dECM suggests the pro‐angiogenic potential of the dECM, including its potential to accelerate angiogenesis and endothelial sprouting.

**FIGURE 5:**
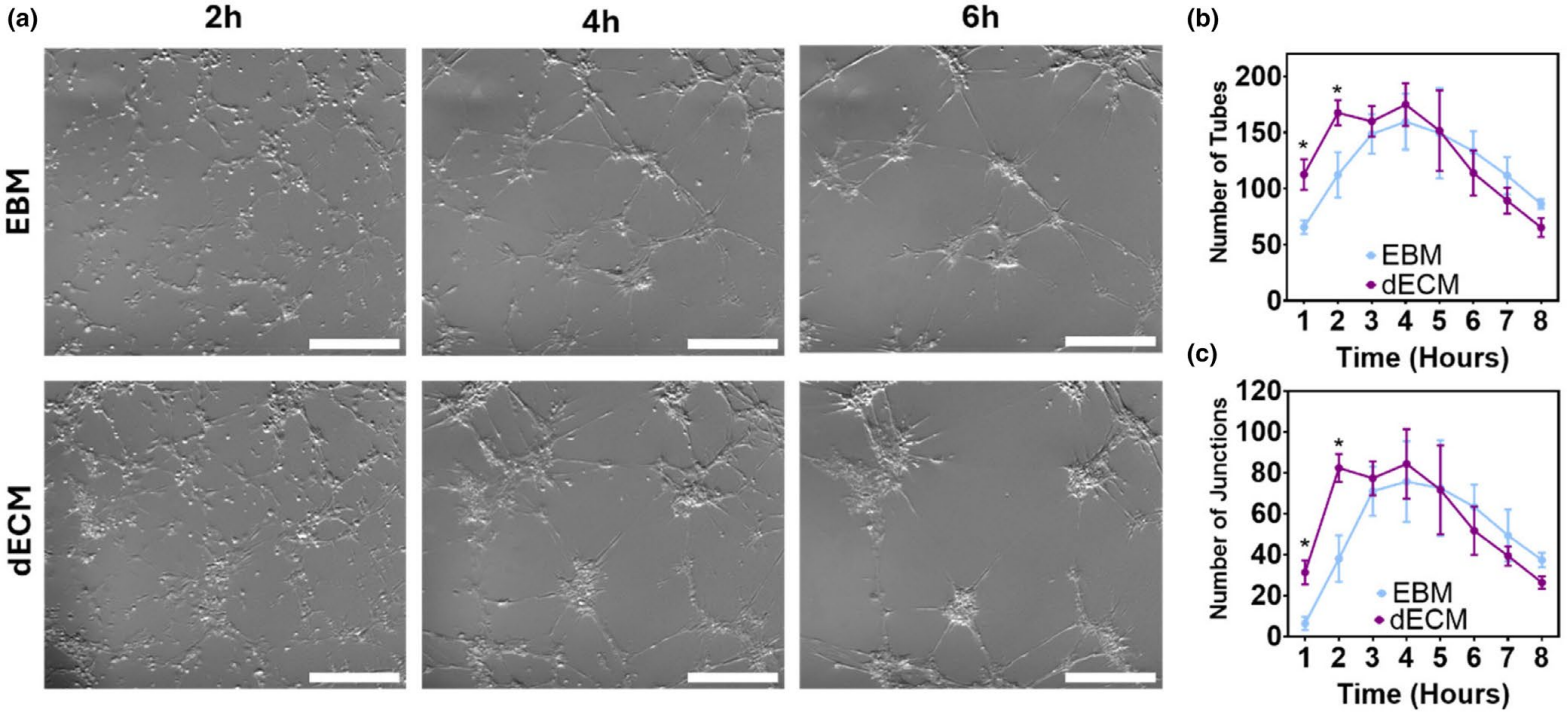
dECM media supplementation accelerates iEC organisation into tube‐like structures. (a) Representative brightfield images show the formation of tube‐like structures by iECs after dECM media supplementation. Scale bar 500 μm. (b) Quantification of the number of tube‐like structures and (c) the number of junctions formed by iECs after dECM supplementation shows the accelerated capacity of cells to organise into tube‐like structures. Data represents mean ± SD; *n* = 3 technical replicates. Analysis performed by two‐way ANOVA with Dunnett's multiple comparisons test.

### 3.6 AcHyA hydrogels can be decorated with dECM without affecting biophysical properties

Following establishment of the pro‐angiogenic capacity of dECM, it was incorporated within a HyA‐based hydrogel (Figure 6a). HyA was chemically modified by the addition of acrylate groups to its backbone (Figure S5). An acrylation efficiency of ~20% was measured by integration of the peaks detected by ^1^H‐NMR (Figure S5C,D). Chemical modification of HyA with acrylate groups allowed for its chemical crosslinking with dithiol crosslinkers, through Michael‐type reaction of thiol and acrylate groups, and the subsequent formation of hydrogels (Browne et al., 2020).

**FIGURE 6:**
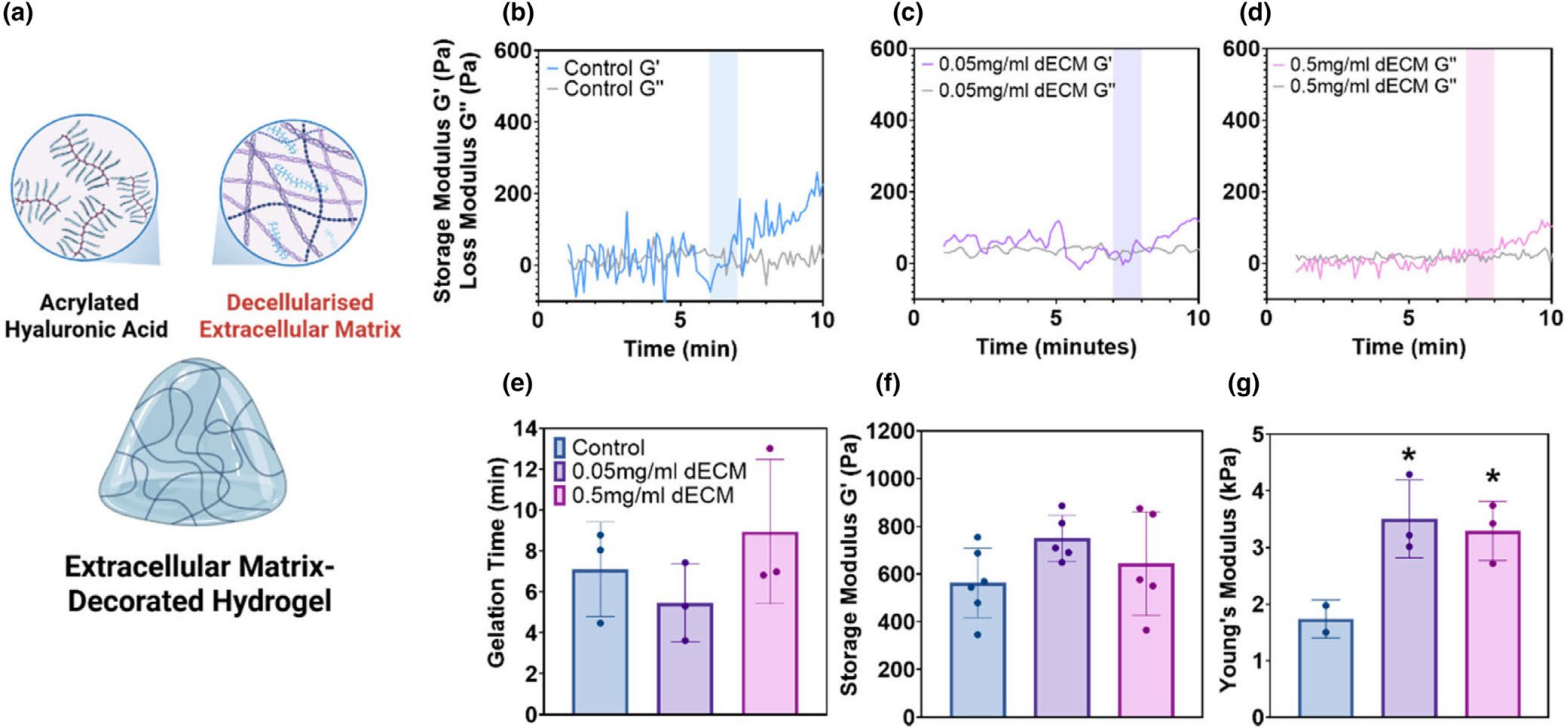
AcHyA hydrogels decorated with dECM have suitable biophysical properties for wound healing. (a) Representative schematic showing the decoration of an AcHyA hydrogel with dECM, to form an ECM‐decorated hydrogel. (b–d) Representative gelation time graphs of AcHyA (control), 0.05 mg/mL dECM and 0.5 mg/mL dECM hydrogels, indicated by crossover of G′ and G″ and marked by shaded area; *n* = 1. (e) AcHyA, 0.05 mg/mL dECM and 0.5 mg/mL dECM hydrogels form stable hydrogels in similar time frames. Gelation time calculated by the point of intersection of the slope of G′ and G″. Data represents mean ± SD and analysed by one‐way ANOVA with Dunnett's multiple comparisons test; *n* = 3 technical replicates. *p < 0.05. (f) Storage modulus (G′) (Pa) of AcHyA, 0.05 mg/mL dECM and 0.5 mg/mL dECM hydrogels indicates no significant difference between each group, *n* = 6. (g) Young's modulus of AcHyA, 0.05 mg/mL dECM and 0.5 mg/mL dECM hydrogels indicates high concentration dECM leads to increased hydrogel stiffness, *n* = 3 technical replicates. Data were analysed by two‐way ANOVA with Dunnett's multiple comparisons test represents mean ± SD, *n* = 3 technical replicates, *(pink): *p* < 0.05 AcHyA (control) versus 0.05 mg/mL dECM.

dECM was solubilised with pepsin and acetic acid for incorporation within HyA‐based hydrogels (Figure 6a). dECM was subsequently added to the AcHyA macromer solution at 0.05 and 0.5 mg/mL, followed by chemical crosslinking with PEG‐dithiol. Gelation time remained unaffected by the addition of dECM to HyA hydrogels, with AcHyA (control), 0.05 mg/mL dECM and 0.5 mg/mL dECM hydrogels forming a stable hydrogel (denoted by storage modulus (G′) exceeding loss modulus (G″)) after 7.1 ± 2.3, 5.5 ± 1.9 and 8.9 ± 3.5 min, respectively (Figure 6b–e).

Storage modulus and Young's modulus were measured across all hydrogels to assess their biophysical properties. AcHyA hydrogels had a storage modulus of 563.4 ± 146 Pa, 0.05 mg/mL dECM hydrogels had storage modulus of 749.4 ± 97.4 Pa and 0.5 mg/mL dECM hydrogels had a storage modulus of 643.7 ± 216.4 Pa. Although there was a trending increase in the storage modulus of 0.05 mg/mL dECM hydrogels, no significant difference was observed (Figure 6f). AcHyA, 0.05 mg/mL dECM and 0.5 mg/mL dECM hydrogels exhibited Young's moduli of 1.7 ± 0.3 kPa, 3.5 ± 0.7 kPa and 3.3 ± 0.5 kPa, demonstrating an increase in compressive modulus following the addition of dECM to AcHyA hydrogels (Figure 6g).

We further measured the swelling ratio to assess the capacity of dECM hydrogels to retain moisture. AcHyA hydrogels swelled to 254.6 ± 17.7% of their original mass, 0.05 mg/mL dECM hydrogels swelled to 246.6 ± 19.3% and 0.5 mg/mL dECM hydrogels swelled to 272.4 ± 17.2% of their original mass (Figure 7a).

**FIGURE 7:**
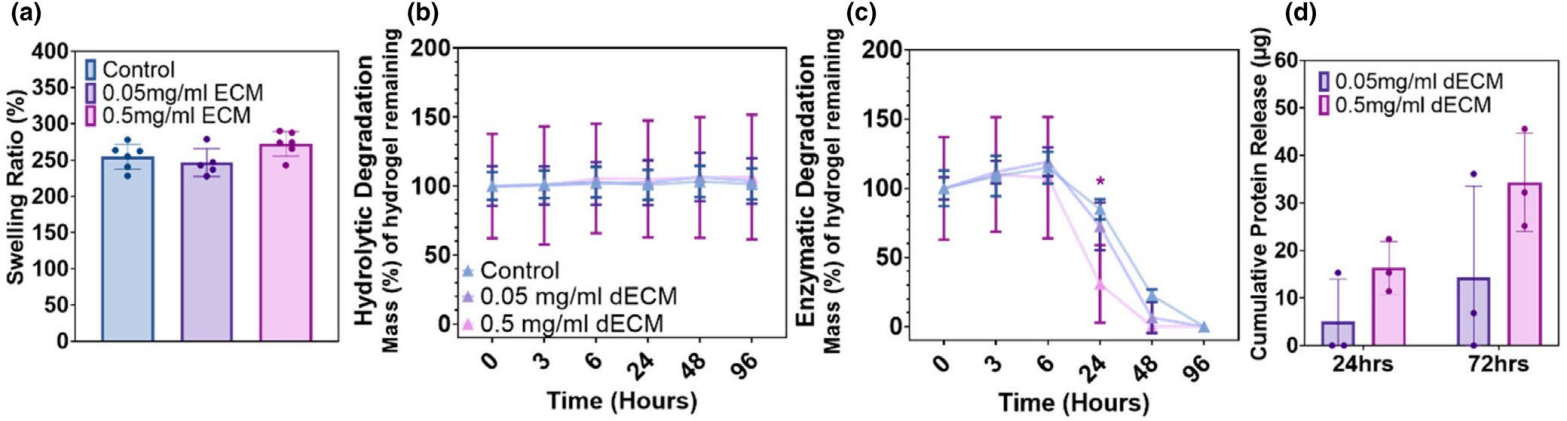
AcHyA hydrogels decorated with dECM have suitable swelling and degradation kinetics for wound healing. (a) Swelling ratio of AcHyA, 0.05 mg/mL dECM and 0.5 mg/mL dECM hydrogels, *n* = 6 technical replicates. (b) Hydrolytic degradation of AcHyA, 0.05 mg/mL dECM and 0.5 mg/mL dECM hydrogels in PBS, after 24 h of swelling in PBS. (c) Enzymatic degradation of AcHyA, 0.05 mg/mL dECM and 0.5 mg/mL dECM hydrogels in 10 U/mL hyaluronidase, after 24 h of swelling in PBS. Data were analysed by two‐way ANOVA with Dunnett's multiple comparisons test represents mean ± SD, *n* = 3 technical replicates, *(pink): *p* < 0.05 AcHyA (control) versus 0.05 mg/mL dECM. (d) Cumulative protein release from 0.05 mg/mL dECM and 0.5 mg/mL dECM hydrogels. Data represents mean ± SD; *n* = 3 technical replicates, with assay replicates in triplicate.

The stability of AcHyA hydrogels, and the impact of dECM incorporation was assessed in hydrolytic and enzymatic conditions. All hydrogel groups remained stable under hydrolytic conditions, swelling to 103.4 ± 11.3% (AcHyA), 106.6 ± 17.6% (0.05 mg/mL dECM) and 106.2 ± 43.7% (0.5 mg/mL dECM) after 48 h in PBS (Figure 7b). In contrast, treatment with hyaluronidase led to complete degradation of all hydrogels after 96 h. The 0.5 mg/mL dECM hydrogels degraded most rapidly, with only 30.8 ± 28% of their mass remaining after 24 h in hyaluronidase, perhaps reflecting the release of dECM after 24 h. In contrast, AcHyA and 0.05 mg/mL dECM hydrogels reached 84.9 ± 7.3% and 72.5 ± 17.2% of their original mass after 24 h, respectively (Figure 7c). However, there was no significant difference in hydrogel mass after 48 h in all three groups. In accordance with this, protein release was measured as a representative of dECM release from the hydrogels. Although there is an apparent trend in increased cumulative protein release in higher concentration dECM‐decorated hydrogels, this remained insignificant (Figure 7d).

### 3.7 The decoration of AcHyA hydrogels with dECM supports pro‐angiogenic‐like behaviour by HUVECs

To determine the pro‐angiogenic potential of the dECM hydrogels, and their capacity to support the growth and activity of cells typically involved in vascularisation, HUVECs and hDFs were seeded on AcHyA (control), 0.05 mg/mL dECM and 0.5 mg/mL dECM hydrogels and metabolic activity assessed over 7 days. Over time, 0.5 mg/mL dECM hydrogels supported a significant increase in HUVEC metabolic activity compared to the control group (Figure 8d), with no increase in hDF metabolic activity detected (Figure 9d). Increased cell coverage per mm^2^ was observed in both dECM‐decorated hydrogel groups, and a trending reduction in cell circularity was observed in HUVECs seeded on 0.5 mg/mL dECM hydrogels (Figure 8e,f). Similarly, hDF cell coverage was significantly higher in 0.5 mg/mL dECM hydrogels, in comparison with both other groups (Figure 9e). Finally, functional assessment of the pro‐angiogenic potential of the dECM‐decorated hydrogels included characterisation of the capacity of conditioned media from hDFs seeded on hydrogels to support the formation of tube‐like structures. Treatment with supernatant from hDFs seeded on 0.5 mg/mL dECM‐decorated hydrogels supported increased total branching length by HUVECs, in comparison with AcHyA (control) hydrogels and 0.05 mg/mL dECM‐decorated hydrogels (Figure 9f–h), demonstrating a dose effect of dECM addition.

**FIGURE 8:**
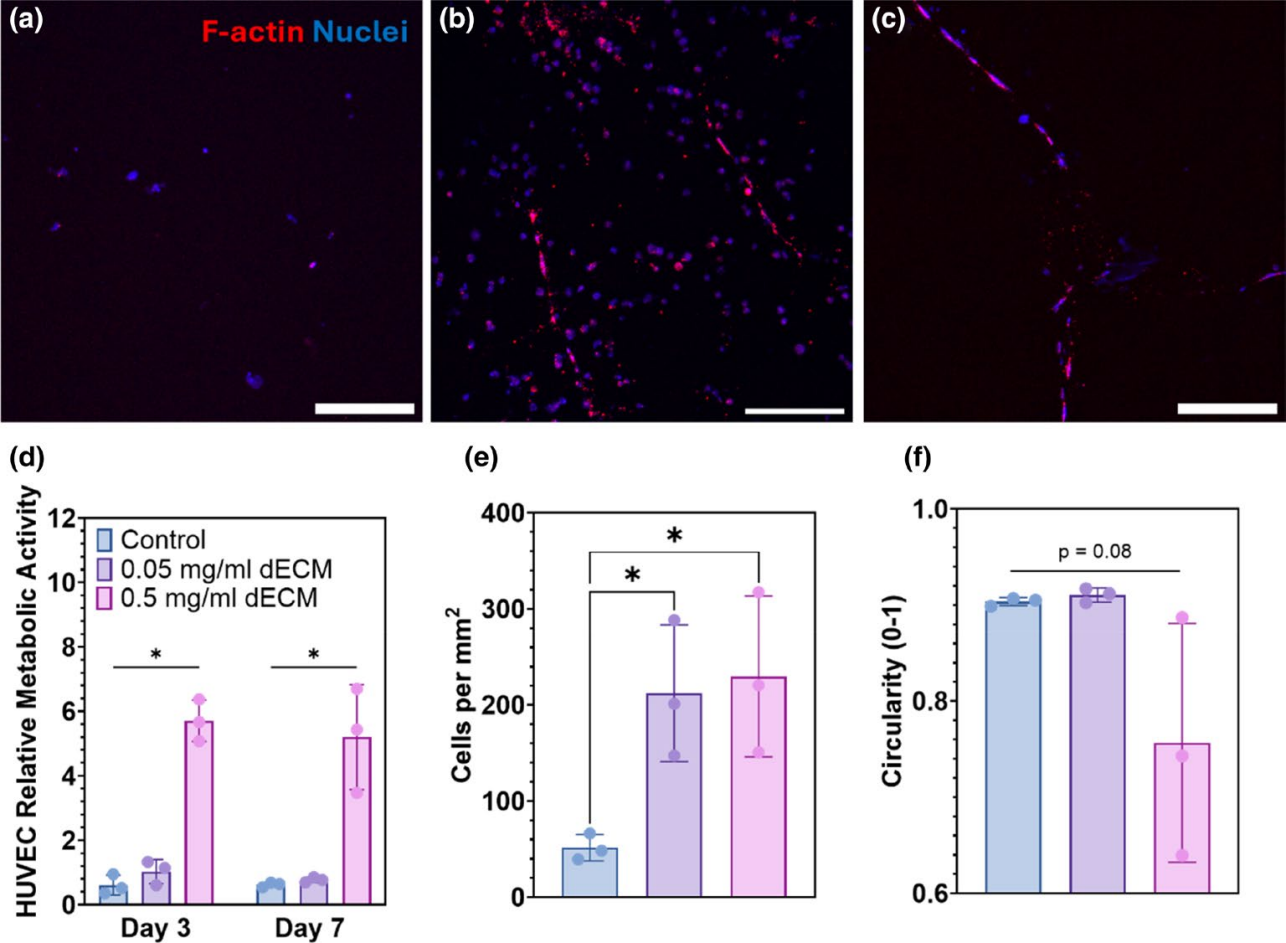
The decoration of AcHyA hydrogels with dECM enhances their pro‐angiogenic capacity by supporting HUVEC metabolic activity and elongation. (a) Immunofluorescent DAPI/F‐actin images of HUVECs seeded on AcHyA, (b) 0.05 mg/mL and (c) 0.5 mg/mL dECM hydrogels after 7 days indicate increased spreading of HUVECs on 0.5 mg/mL dECM hydrogels. Scale bar 200 μm. (d) Relative metabolic activity of HUVECs seeded on AcHyA (control), 0.05 mg/mL and 0.5 mg/mL dECM hydrogels over 7 days, *n* = 3 technical replicates with assay replicates in triplicate. Data analysed by two‐way ANOVA with Dunnett's multiple comparisons test. (e) Quantification of cells attached per mm^2^, average Feret diameter and cell circularity on AcHyA, 0.05 mg/mL dECM and 0.5 mg/mL dECM hydrogels. Data analysed by one‐way ANOVA with Tukey's multiple comparisons test. *n* = 3 technical replicates, with imaging replicates in triplicate. *p < 0.05; data represents mean ± SD.

**FIGURE 9:**
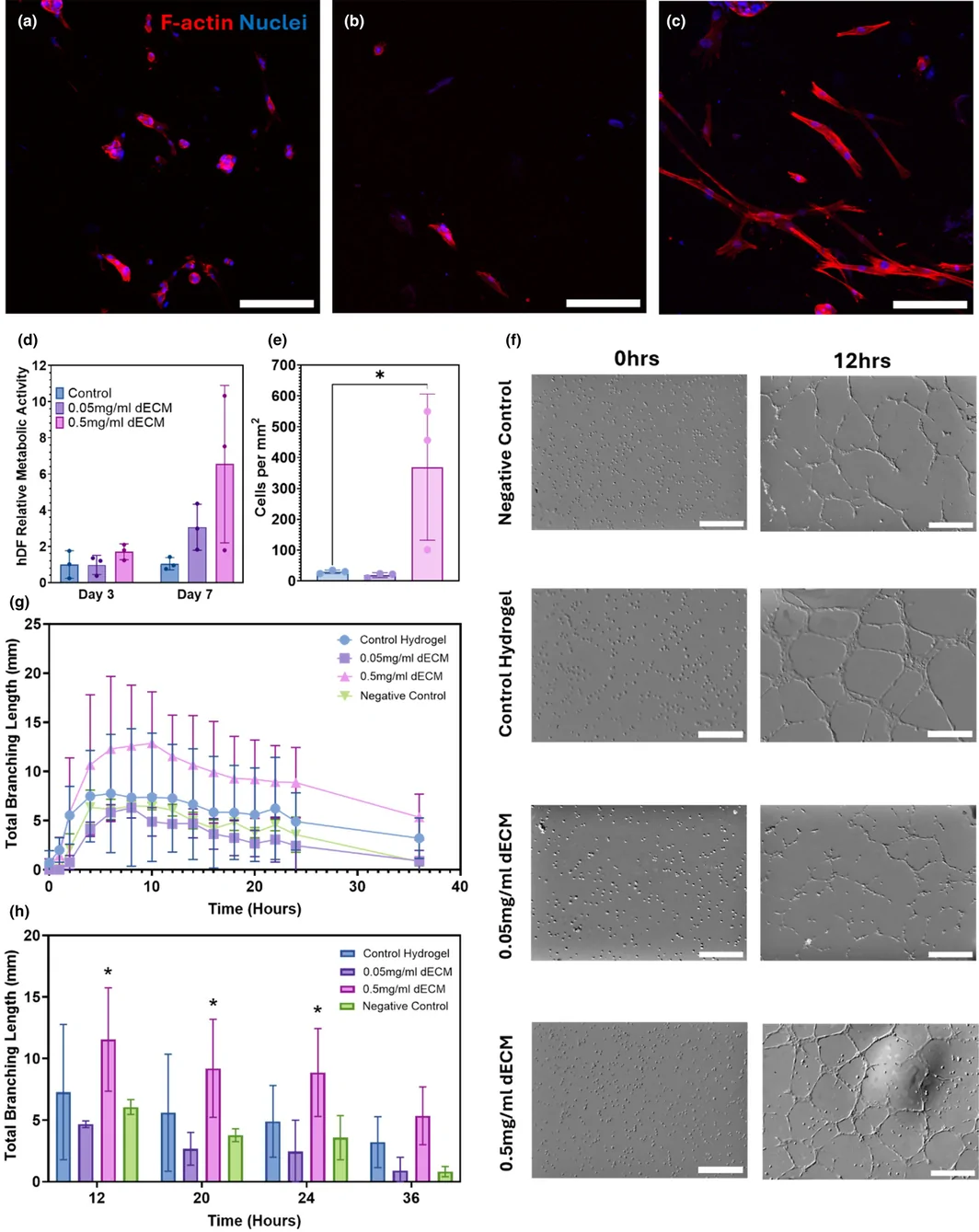
The decoration of AcHyA hydrogels with dECM enhances their pro‐angiogenic capacity by supporting hDF metabolic activity and elongation, and the formation of tube‐like structures. (a) Immunofluorescent DAPI/F‐actin images of hDFs seeded on AcHyA, (b) 0.05 mg/mL and (c) 0.5 mg/mL dECM hydrogels after 7 days indicates increased spreading of hDFs on 0.5 mg/mL dECM hydrogels. F‐actin staining indicated by arrows. Scale bar 200 μm. (d) Relative metabolic activity of hDFs seeded on AcHyA (control), 0.05 and 0.5 mg/mL dECM hydrogels over 7 days, *n* = 3 technical replicates with assay replicates in triplicate. Data represent mean ± SD. Data analysed by two‐way ANOVA with Dunnett's multiple comparisons test. (e) Quantification of cells attached to each hydrogel per mm^2^, *n* = 3 technical replicates with imaging replicates in triplicate. Data represent mean ± SD, and analysed by one‐way ANOVA with Tukey's multiple comparisons test. *p< 0.05. (f) Representative brightfield images of tube formation assay, comparing the capacity of conditioned media from hDFs seeded on control, 0.05 mg/mL dECM and 0.5 mg/mL dECM hydrogels to support the formation of tube‐like structures. (g, h) Quantification of total branching length over 36 h. Data represent mean ± SD. Data analysed by two‐way ANOVA with Tukey's multiple comparisons test. *p < 0.05 compared to control hydrogels and 0.05 mg/mL dECM hydrogels. *n* = 3 assay replicates.

## 4 DISCUSSION

Chronic wounds present a global healthcare concern due to their non‐healing nature. Current treatments remain insufficient, with poor vascularisation within the wound bed a major inhibitor of tissue repair and healing. As such, there is a need for the development of new strategies to drive angiogenesis and re‐vascularisation to support wound closure and tissue regeneration. In the present study, we show that hiPSC‐derived vascular cell‐deposited ECM contains components which play a role in wound healing, and the promotion of angiogenesis during healing. We have also shown that post‐decellularisation, this dECM can drive pro‐angiogenic processes in vitro, including the transient acceleration of the formation of tube‐like structures in 2D, in addition to enhanced metabolic activity and attachment of both endothelial cells and fibroblasts to HyA‐based dECM‐decorated hydrogels in 3D. Similarly, conditioned media from fibroblasts seeded on dECM‐decorated hydrogels enhanced the formation of vascular‐like structures, indicating the capacity of dECM‐decorated hydrogels to drive pro‐angiogenic behaviour of fibroblasts. Taken together, these results demonstrate that hiPSC‐derived vascular cell‐deposited ECM enhances the interactions between endothelial cells, fibroblasts and AcHyA hydrogels to support pro‐angiogenic behaviour. Through the establishment of a pro‐angiogenic microenvironment, these hydrogels have the potential to facilitate the infiltration and spreading of endothelial cells throughout the wound bed, thus stimulating re‐vascularisation and healing of chronic wounds.

To develop pro‐angiogenic ECM, hiPSCs were differentiated towards a vascular lineage, yielding a heterogenous population of CD31 expressing iECs, and CD31‐iSCs. The present study employed hiPSC‐derived vascular cells for several reasons. In comparison with primary somatic cells or cell lines, their fetal‐like nature, reduced variability and fewer ethical concerns make them an attractive source of ECM (Moradi et al., 2019). Similarly, in addition to their increased expansion capacity and suitability for scale‐up, hiPSC‐derived cells have demonstrated the ability to deposit ECM that drives tissue vascularisation and resolution of inflammation. This capacity was demonstrated by the reduction in tumour necrosis factor‐α (TNF‐α) production by macrophages, and increased VEGF secretion by diabetic fibroblasts seeded on hiPSC‐derived cell‐deposited dECM‐functionalised scaffolds (Santarella et al., 2020). Similarly, iPSC‐derived fibroblasts have an increased capacity to deposit ECM in comparison with their somatic counterparts (Huang et al., 2025; Shamis et al., 2012). Furthermore, mesenchymal stromal cells (MCS) cultured on iPSC‐derived MSC‐deposited dECM had enhanced osteogenic, chondrogenic and adipogenic potential in comparison with dECM deposited by bone marrow derived stromal cells and a placenta‐derived MSC cell line (Huang et al., 2025). The capacity of ECM deposited from hiPSC‐derived cells to enhance healing in vivo to a greater extent than primary cells has also been reported. For example, increased deposition of collagens was observed in hiPSC‐derived endothelial cell‐treated murine wounds, in addition to accelerated wound perfusion and closure (Clayton et al., 2018). In the present study, we differentiated hiPSCs from a single male donor to show the potential of hiPSC‐derived vascular cells to deposit pro‐angiogenic ECM. Although the data presented here is promising, it is important to acknowledge the effect donor variability can have, and how this may influence the outcome. Donor variability can occur as a result of many factors, including sex, genetic background and reprogramming instability (Vitale et al., 2012). In particular, the genetic background of hiPSC donors has shown to be the largest contributor to variability, by analysis of hiPSC‐derived astrocyte proteome (Beekhuis‐Hoekstra et al., 2021). In the context of the present study, this variability could lead to differences in ECM yield, composition and ultimately the activity of the hydrogel. Future studies incorporating multiple hiPSC donor lines from different sex and genetic backgrounds are necessary to verify the extent to which ECM deposition and composition are affected by the use of different hiPSC lines.

After separation of iECs and iSCs by MACS, the iSC population was characterised to identify the cell types present. Immunofluorescent staining for SM‐22α and vimentin indicated the presence of markers of several cell types, for example, pericytes, smooth muscle cells and fibroblasts. The heterogeneity of the iSC population shown here makes it an ideal source of pro‐angiogenic ECM. It has been shown previously that co‐cultures of hiPSC‐derived endothelial cells and smooth muscle cells increased neovascularisation both in 2D culture and in a murine wound model, in comparison with primary cells (Kim et al., 2013). Similarly, the presence of several key supportive cell types supports the deposition of structurally supportive ECM components, ultimately allowing for the development of functionally mature vascular structures (Nehls et al., 1992; Sukmana & Vermette, 2010; Velazquez et al., 2002). As such, iSCs were identified as most suitable for the deposition of ECM in the present study.

ECM deposition by iSCs was enhanced by the addition of ascorbic acid (AA) and macromolecular crowder carrageenan (C) to the cell culture media. Macromolecular crowding is an established technique that drives increased ECM deposition in cell culture settings (Satyam et al., 2014; Zeugolis, 2021). Through the addition of an inert macromolecule to culture media, in this case carrageenan, macromolecular diffusion in the microenvironment is reduced due to steric hindrance, thus accelerating biochemical processes including ECM deposition (Zeugolis, 2021). AA was included in the media to enhance ECM deposition, due to its role as a co‐factor in the hydroxylation and subsequent deposition of collagens (Murad et al., 1981). Similarly, carrageenan was selected as the MMC in this study as it has been previously shown to increase ECM deposition in comparison with other crowders, such as dextran sulphate, in shorter time frames. This includes increased deposition of collagens, laminins and fibronectin, likely due to its high polydispersity index (Gaspar et al., 2019; Satyam et al., 2014). In the present study, the addition of AA and carrageenan to the culture media enhanced the deposition of ECM by iSCs over 21 days, allowing for the decoration of AcHyA hydrogels, as presented here. However, further optimisation and scale‐up of ECM deposition is necessary to increase ECM yield to a level which would be sufficient for clinical translation. Another barrier to clinical translation is the presence of animal products in the media, specifically 2% fetal calf serum present in EGM‐2. This may raise issues in the context of both batch‐to‐batch variation and immunogenicity. Future optimisation of ECM deposition should include the use of serum replacements or defined media free of animal‐derived products.

Efficient decellularisation of ECM is essential as insufficient removal of cellular content or DNA can lead to adverse immune responses. The presence of cellular material within non‐decellularised ECM has the capacity to polarise macrophages, thus stimulating the onset of an M1 pro‐inflammatory macrophage response, and the formation of scar tissue (Brown et al., 2009). To avoid adverse immune responses, Crapo et al. identified some criteria including: (i) <50 ng dsDNA/mg ECM dry weight, (ii) DNA fragments of <200 base pairs and (iii) negative DAPI or H&E staining (Crapo et al., 2011). As such, a critical aim of this study was the optimisation of the decellularisation protocol. Initially, our protocol included a combination of various chemical treatments; however, no effect on DNA concentration was observed. After the addition of enzymatic (DNAse) and mechanical (F/T) decellularisation steps, we observed a significant decrease in DNA content in all treatment groups. Similar to surfactants like triton X‐100, F/T causes cell lysis and the dissociation of cell membranes from the ECM (Zhang et al., 2022), and has been shown to be particularly effective in combination with triton X‐100 and deionised water (Aeberhard et al., 2020). The significant reduction in DNA concentration observed here is critical to avoid unwanted and adverse host responses.

Critical aims of the present study included verifying the presence of deposited ECM components, ensuring their retention post‐decellularisation and characterisation of their pro‐angiogenic nature. Immunofluorescent and histological staining were used to characterise the deposited ECM, and to assess the effect of decellularisation on ECM retention. ECM components which play critical roles in the early phases of wound healing, and those with pro‐angiogenic properties were identified for staining. Here we have shown that iSCs deposit a range of ECM components integral to tissue vascularisation and wound healing, including fibronectin, fibrillar collagens, perlecan, GAGs and sGAGs. Shortly after injury to healthy skin, fibronectin rapidly replaces the fibrin clot, providing biophysical cues to endothelial cells for the initiation of angiogenesis through the presentation of integrin binding sites. This stimulates both endothelial cell sprouting and the formation of tube‐like structures (Tonnesen et al., 2000). Similarly, collagen III is deposited within the wound by fibroblasts shortly thereafter, followed by its replacement by collagen I (Tonnesen et al., 2000). These fibrillar collagens play important roles in the progression of wound healing and angiogenesis, particularly in actin cytoskeleton organisation, endothelial cell survival and structural support (Mongiat et al., 2016). Perlecan is a heparan sulphate proteoglycan, found in the basement membrane. Its presence in iSC‐deposited ECM is particularly notable as it has the capacity to interact with, and potentiate the effect of a number of pro‐angiogenic growth factors (Kim et al., 2023). VEGF and fibroblast growth factor‐2 (FGF‐2), for example, interact with heparan sulphate chains on Domain I of perlecan, allowing for their sequestration, protection from degradation and presentation to endothelial cells (Bix & Iozzo, 2008). Similarly, GAGs in general, for example, chondroitin sulphates, hyaluronic acid, heparan sulphates and keratan sulphates, were detected by alcian blue. Their roles in the formation and repair of blood vessels also include growth factor and cytokine binding, and the regulation of endothelial cell proliferation during vasculogenesis (Lin et al., 2025). The presence of sGAGs, as detected by Alcian blue staining, may also further enhance the wound healing/angiogenic capacity of the dECM. This has recently been shown through the decellularisation of various perinatal tissues, and assessment of their reparative capacity in the context of GAGs. It was found that placenta‐dECM had a naturally higher content of sGAGs, and when used to treat murine wounds in the form of hydrogels, stimulated enhanced expression of anti‐inflammatory and pro‐angiogenic genes (Wang et al., 2021). Similarly, collagen scaffolds functionalised with GAGs such as chondroitin‐6‐sulphate and laminin molecules enhanced VEGF production by fibroblasts, further showing their pro‐angiogenic potential (McGrath et al., 2026). Although several components were well retained after decellularisation, the low level of alcian blue staining must be noted. A commonly reported disadvantage of chemical decellularisation agents is the loss of GAGs from the ECM (Gilbert et al., 2006). However, it is also important to note that GAGs/sGAGs detected by alcian blue staining are not fully representative of extracellular GAGs, due to their presence on the cell membrane (Linhardt & Toida, 2004). As such, the alcian blue staining shown in the present study signifies that through decellularisation, the apparent loss of GAGs/sGAGs may signify the loss of cellular GAGs, and the retention of those deposited by iSCs in the extracellular space. Furthermore, the work presented here involves the addition of the dECM to a non‐sulphated GAG (HyA)‐based hydrogel, which mitigates the low GAG deposition. Collectively, the presence of fibronectin, fibrillar collagens, perlecan, GAGs and sGAGs demonstrates the pro‐angiogenic nature of dECM.

The pro‐angiogenic nature of the dECM was confirmed in 2D by the acceleration of the formation of tube‐like structures by iECs, and was subsequently incorporated into hydrogels. EBM was chosen as a negative control in this instance, due to its lack of growth factors which typically stimulate the vascular‐like organisation of endothelial cells. Thus, it allowed us to effectively isolate the pro‐angiogenic potential of the dECM from the media composition. We modify HyA with acrylate groups for the development of HyA‐based hydrogels (Browne et al., 2018; Dienes et al., 2021; Petit, Chang, et al., 2025; Rohrer et al., 2024). This approach allows for chemical crosslinking with PEG‐dithiol, and the subsequent in situ formation of hydrogels, as well as the possibility to add additional bio‐functionality through the conjugation of peptides or other bioactive ligands bearing a thiol moiety (Petit, Gomes, et al., 2025). In the present study, we used 500 kDa molecular weight HyA, which has previously been shown to have properties permissive to vascularisation (Browne et al., 2020). Furthermore, we used both low (0.05 mg/mL) and high (0.5 mg/mL) concentrations of dECM for the decoration of AcHyA hydrogels to enhance their pro‐angiogenic potential. Typically, dECM is used at higher concentrations when it is used to form dECM hydrogels without the need for any additional polymer components. However, in the present study, the concentrations were selected to provide bioactive cues to an AcHyA hydrogel without effecting its biophysical properties. Future studies may focus on the further optimisation of dECM concentration to further enhance cellular attachment and to fine‐tune the pro‐angiogenic cellular behaviour. Importantly, we found that the addition of dECM to AcHyA hydrogels did not compromise their biophysical properties. This includes maintenance of storage modulus and swelling ratio, and increased Young's modulus. Although high concentration dECM hydrogels demonstrated accelerated degradation within the first 24 h compared to AcHyA hydrogels. The degradation kinetics stabilised after 48 h and followed a similar trajectory thereafter. Similarly, the slower gelation time of high concentration ECM hydrogels may be a result of steric hindrance between PEG and AcHyA, with reduced accessibility and interaction between dithiol/acrylate groups for crosslinking. It is important to note that in this study, dECM was mixed with the hydrogel pre‐gelation, and was not chemically conjugated to the AcHyA system. As we have shown in Figure 7(d), dECM was gradually released into the supernatant as the AcHyA‐based hydrogel degraded. As a result, this released dECM can stimulate pro‐angiogenic behaviour by surrounding cells in the wound bed, supporting vascularisation and healing. Furthermore, as cells remodel and degrade the AcHyA‐based hydrogels, the altered biophysical properties are expected to increase cellular infiltration through durotaxis, leading cells to migrate along gradients of mechanical stiffness (Isomursu et al., 2022).

Incorporation of dECM into AcHyA hydrogels also supported angiogenic behaviour of both endothelial cells and fibroblasts. This was observed in a dose‐dependent manner, with high concentration dECM supporting increased metabolic activity and the attachment and elongation of HUVECs. HUVEC elongation and spreading is typically seen during the formation of vascular networks and tube‐like structures in vitro (Merks et al., 2006). Similar behaviour was observed by fibroblasts, which showed increased cell attachment to 0.5 mg/mL dECM hydrogels. We also observed the pro‐angiogenic‐like nature of dECM‐decorated hydrogels through treatment of HUVECs with the hDF secretome and their increased tendency towards vascular‐like organisation in a concentration‐dependent manner. The lack of effect observed at low concentration dECM suggests a dose‐effect of dECM and that lower concentration dECM provides insufficient signals, such as cell adhesive ligands (D'Souza et al., 1991; Knight et al., 1998) and growth factor sequestering moieties to support cell attachment and activity (Zhou et al., 2004).

Taken together, the present study describes the development and in vitro assessment of a hiPSC‐derived vascular cell‐deposited dECM hydrogel, which has the capacity to enhance endothelial cell activity and to support the formation of tube‐like structures. We confirmed the presence of some key ECM components pre and post‐decellularisation, which play integral roles in angiogenesis and healing. Future work will focus on further assessment of the pro‐angiogenic capacity of the dECM, in‐depth characterisation of its composition and ultimately its assessment in a preclinical wound model to determine its ability to drive wound healing.

## 5 CONCLUSION

The present study describes the development of a promising HyA‐based hydrogel for the treatment of chronic wounds. Owing to their supportive function and vascular origin, iSCs deposited an ECM containing components such as fibronectin, collagens, perlecan and GAGs. Treatment of endothelial cells with this dECM in 2D allowed for the transient acceleration of the formation of tube‐like structures. Additionally, this dECM facilitated enhanced metabolic activity, attachment and spreading of both HUVECs and hDFs when seeded on dECM‐decorated hydrogels at high concentrations. We demonstrate that hiPSC‐derived vascular cell‐derived ECM combined with HyA‐based hydrogels is a promising approach for the potential stimulation of angiogenesis.

## AUTHOR CONTRIBUTIONS

**Louise Hosty:** data curation, formal analysis, investigation, methodology and writing—original draft. **Yu‐Yin Joanne Chang:** data curation, formal analysis, investigation, methodology and writing—review and editing. **Martina Konečná:** data curation, formal analysis, investigation, methodology. **Matthew McGrath:** data curation, formal analysis, investigation, methodology. **Cormac Murphy:** data curation, formal analysis, investigation, methodology. **Anja Schweikert:** data curation, formal analysis, investigation, methodology. Mari Ozaki: generation and characterisation of the RCSI‐D1 iPSC line. **Killian Hurley:** methodology and resources. **Fabio Quondamatteo:** conceptualisation, supervision, funding acquisition, and writing—review and editing. **Shane Browne:** conceptualisation, supervision, funding acquisition, and writing—review and editing.

## Supporting information


Figure S1.

## Data Availability

The data that support the findings of this study are available from the corresponding author upon reasonable request.
