# Supplementary material for: Development of a pro‐angiogenic stem cell‐derived vascular cell extracellular matrix‐decorated hyaluronic acid hydrogel
Source: J Anat. 2026 Nov 4:10.1111/joa.70273. Online ahead of print. doi: 10.1111/joa.70273 (PMC13736179; doi:10.1111/joa.70273)
Supplement: Supplementary file 1 [file JOA-9999-0-s001.docx]

**Development of a Pro-Angiogenic Stem Cell-Derived Vascular Cell Extracellular Matrix-Decorated Hyaluronic Acid Hydrogel**

Louise Hosty^1^, Yu-Yin Joanne Chang^1, 2^, Martina Konečná^1^, Matthew McGrath^1^, Cormac Murphy^1,4^, Anja Schweikert^1,4^, Mari Ozaki^1,4^, Killian Hurley^1,4^, Fabio Quondamatteo^1^, Shane Browne^1, 2, 3^

*1. Tissue Engineering Research Group, Department of Anatomy and Regenerative Medicine, Royal College of Surgeons in Ireland, Dublin, Ireland*

*2. CÙRAM, Centre for Research in Medical Devices, University of Galway, Galway, Ireland*

*3. Trinity Centre for Biomedical Engineering, Trinity College Dublin, Dublin, Ireland*

*4. Department of Medicine, Education and Research Centre, Royal College of Surgeons in Ireland, Dublin, Ireland*

**Supplementary Materials**

***Results***

**Figure S1**

**Figure S2**

**Figure S3**

**Figure S4**

**Figure S5**

**Figure S6**

***Figure Legends***

**Figure S1. Macromolecular crowding supplementation of carrageenan (C) and ascorbic acid (AA) does not negatively affect iSC viability**. A. PicoGreen assay to determine the DNA concentration, as a measure of cell proliferation; n=3. B. AlamarBlue assay measuring metabolic activity of iSCs over 21 days of AA, or AA+C treatment; n=6. Data represents mean ± SEM. Analysis performed by 2-way ANOVA with Dunnet’s multiple comparisons test; * = p<0.05. (C-D) Brightfield images of iSCs after 21 days of AA, AA+C supplementation.

**Figure S2. Treatment of iSCs with 0.1% triton X-100, ammonium hydroxide, and deionised water had no effect on DNA concentration or DAPI staining.** A. Quantification of DNA concentration in non-decellularised and decellularised samples after 21 days of supplementation with AA or AA+C. B. DAPI staining of cell nuclei in non-decellularised and decellularised samples after 21 days of supplementation with AA or AA+C. Scale bars 100μm. Data represents mean ± SEM. Analysis performed by 2-way ANOVA with Sìdàks multiple comparisons test; n=3, * = p<0.05 in comparison to corresponding non-decellularised sample.

**Figure S3. ECM yield is highest when cells are supplemented with AA+C, 2% serum, and at 13,000 cells/cm^2^.** A. Effect of macromolecular crowding on ECM yield in comparison to EGM-2; n=6. B. Effect of lower iSC seeding density on ECM yield, in comparison to 13,000 cells/cm^2^; n=3. C. Effect of lower culture media serum concentration on ECM yield, in comparison to standard EGM-2 (2%); n=3. Data represents mean ± SEM. Analysis performed using one-way ANOVA with Dunnett's multiple comparisons test. * = p<0.05.

**Figure S4. iSC-ECM contains fibronectin, fibrillar collagens, GAGs, and sGAG**. A. Immunofluorescent and histological staining of ECM and deposited by control iSCs over 21 days. B. Immunofluorescent and histological staining of ECM and dECM deposited by iSCs supplemented with AA over 21 days.

**Figure S5. Hyaluronic acid (HyA) is functionalised with acrylate groups with an efficiency of approximately 20%.** A. Chemical structure of hyaluronic acid (HyA) and (B) acrylated hyaluronic acid (AcHyA). C. NMR spectra of hyaluronic acid, indicated by the peak at a (blue). D. NMR spectra of acrylated hyaluronic acid, with acrylate groups indicated by peak at b (red), showing ~20% acrylation efficiency.

**Figure S6. dECM hydrogels support HUVEC spreading after 3 days.** A. Representative brightfield images of HUVECs seeded on AcHyA (control), 0.05mg/ml dECM, and 0.5mg/ml dECM hydrogels after 3 days.
